# Peripheral Artery Disease: Why Do We Need CT Imaging?

**DOI:** 10.5334/jbsr.3385

**Published:** 2024-04-17

**Authors:** Thomas Jardinet

**Affiliations:** 1 UZA, BE

**Keywords:** Peripheral arterial disease, CT Angiography, Revascularization, Endovascular treatment

## Abstract

The most common cause of peripheral arterial disease (PAD) is atherosclerosis. PAD can be considered a global pandemic, as it affected a quarter billion people worldwide in 2013 [1]. The prevalence and complexity of the disease is increasing due to aging populations and the rise in risk factors including diabetes and kidney disease.

## Clinical Manifestations

The severity of symptoms depends on the extent of the disease, activity level of the patient and the presence of collateral vessels. Most patients are asymptomatic, and routine screening is not typically performed. When symptoms occur, these are related to demand ischemia and manifest as claudication. A subgroup of patients may evolve to more severe symptoms such as rest pain, nonhealing skin ulcerations or gangrene indicating chronic critical limb ischemia (CLI). This condition is associated with a 25% one-year mortality rate and 25% one-year amputation rate [2].

## Diagnostic Work Up

The measurement of segmental systolic blood pressures and ankle-brachial pressure index (ABI) is crucial in the work-up of PAD. An ABI below 0.9 indicates the existence of PAD. In patients with heavily calcified, non-compressible vessels, ABI is not always reliable, and a toe-brachial index (TBI) can be used as an alternative. Transcutaneous oximetry plays an additional role in assessing skin vascularization in CLI [3].

## Treatment Approaches

Treatment of claudication must include medical therapy, risk factor modification, and exercise therapy. Controlled muscle ischemia caused by sufficiently intense exercise stimulates angiogenesis and collateral formation, thus improving PAD symptoms [4].

Surgical or interventional revascularization procedures should be considered in all CLI patients and in patients suffering from persistent debilitating claudication despite maximal medical therapy, risk factor control and at least three months of exercise therapy. Surgical, endovascular, or hybrid approaches can be used, and the choice depends on patient risk, symptom severity, and the exact anatomical pattern of the disease [5].

## Role of Radiologists

Radiologists play a pivotal role in all PAD patients eligible for revascularization. Detailed anatomical images of the arterial system are essential for determining the extent of the disease and for planning of revascularization procedures. Complete pre-procedural assessment by the radiologist has the potential to improve decision making and patient outcomes. Computed tomography angiography (CTA) is often used, since the vessel wall can be well assessed in addition to the vessel lumen. Especially the amount of calcification can determine the choice of treatment in selected cases.

## Level of the Disease

The level of PAD can be divided into inflow disease, outflow disease, and runoff disease. Inflow vessels consist of the aortoiliac arteries including the common femoral artery (CFA) and the origin of the deep femoral artery (DFA). Outflow starts from the origin of the superficial femoral artery (SFA) to the trifurcation of the popliteal artery. Runoff vessels include the anterior tibial artery, tibiofibular trunk, posterior tibial artery, and the fibular artery.

Inflow arteries are more commonly affected in smokers and hypercholesterolemia patients, while age and diabetes are risk factors associated with predominant outflow and runoff disease.

## Revascularization Strategies

In patients with combined in- and outflow disease, the decision to perform staged versus multilevel revascularization is based on the severity of tissue loss, anatomical complexity and patient risk. In patients with claudication, rest pain, or even minor tissue loss, inflow correction can sometimes sufficiently raise the pressure in collateral vessels arising from the deep femoral artery to achieve the desired outcome [6].

In patients with larger wounds or gangrene, the goal is immediate establishment of in-line flow from the aorta to the foot, by at least one well-patent runoff vessel. Most patients with aortoiliac disease can be treated using an endovascular approach. Surgery is reserved for extensive occlusions or after failure of the endovascular option.

For CFA lesions, which often contain bulky calcifications, open endarterectomy, or short interposition grafting remains the standard treatment. Providing sufficient inflow in the DFA is an important component of the surgery. In recent years, some centers have used super flexible nitinol stents as an alternative to open surgery in CFA disease.

Regarding infra-inguinal revascularization, bypass surgery and endovascular therapy have complementary roles and the choice is made based on severity of limb symptoms and anatomical complexity. In general, bypasses are preferred in patients with high anatomical complexity and severe symptoms and when a suitable vein is present, while endovascular treatment is preferred in less anatomically complex cases. Autologous venous bypasses from the greater saphenous vein (GSV) perform far better than venous allografts or prosthetic conduits [5]. The suitability of the GSV depends on the diameter on different levels, which is usually assessed by Doppler ultrasound, but it can also be evaluated on computed tomography (CT) angiography [7].

In candidates for infra-inguinal bypass surgery, accurate imaging and reporting of popliteal and infrapopliteal disease is important since these define the location of the distal anastomosis, which can be in the proximal popliteal artery (P1), in the distal popliteal artery (P3), or even in runoff vessels.

Revascularization procedures of runoff vessels are a subject of debate. When considered, accurate imaging is of uttermost importance. However, this can be challenging in these relatively small-sized arteries compared to the spatial resolution. In elderly people as well as in patients with diabetes or chronic kidney disease, image quality of CTA below the knee can be impaired by blooming artefacts caused by extensive, diffuse, circumferential vessel wall calcifications. These calcifications are distinct from atherosclerosis. They arise from the medial arterial wall and are caused by imbalance in calcium homeostasis [8].

Technical advancements such as photon-counting CT have the potential to overcome these limitations in arteries below the knee [9].

## Conclusion

The main goal of CT angiography is to guide revascularization therapy, which is only indicated in CLI patients and in claudication patients with failure of conservative therapy. Accurate imaging of the extent of occlusive disease is critical in treatment planning. Inflow (including CFA and DFA), outflow and runoff disease should be regarded as separate problems since they are usually treated in different stages, except in severe CLI patients.
